# Hemoglobin Derived from Subarachnoid Hemorrhage-Induced Pyroptosis of Neural Stem Cells via ROS/NLRP3/GSDMD Pathway

**DOI:** 10.1155/2023/4383332

**Published:** 2023-01-16

**Authors:** Tingting Yue, Xiaojian Li, Xiangxin Chen, Ting Zhu, Wei Li, Bin Wang, Chunhua Hang

**Affiliations:** ^1^Department of Neurosurgery, Nanjing Drum Tower Hospital, The Affiliated Hospital of Nanjing University Medical School, Nanjing 210008, China; ^2^Nanjing Drum Tower Hospital Clinical College of Nanjing University of Chinese Medicine, Nanjing 210008, China; ^3^Clinical Stem Cell Center, Nanjing Drum Tower Hospital, The Affiliated Hospital of Nanjing University Medical School, Nanjing 210008, China

## Abstract

Accumulating evidence has demonstrated that neural stem cells (NSCs) have regenerative capacity after brain injuries, such as in aneurysmal subarachnoid hemorrhage (SAH). The reactive oxygen species (ROS)-induced NOD-like receptor thermal protein domain associated protein 3 (NLRP3) inflammasome triggers inflammatory responses and pyroptosis of cells; however, whether ROS-induced neuroinflammation modulates the fate of endogenous NSCs after SAH remains largely unknown. In this study, the level of IL-1*β* was increased in the cerebrospinal fluid (CSF) of patients with SAH. In an endovascular perforation model of SAH in mice, the secretion of IL-1*β* increased to a peak at 24 h following SAH, and the expression of Caspase1 and NLRP3 was elevated in the hippocampus. Primary cultured NSCs were incubated with hemoglobin (Hb) to mimic SAH in vitro. The cell viability, LDH release, intracellular ROS levels, scanning electron microscopy (SEM), and the expression of NLRP3 and pyroptosis indicators (GSDMD, ASC, and Caspase-1) in NSCs after SAH were examined to investigate the process of pyroptosis. We found that pyroptotic death featuring cellular swelling, cell membrane pore formation and elevated IL-1*β* was increased in cultured primary NSCs after Hb treatment, as was the expression of NLRP3, ASC, Caspase-1, and GSDMD. In addition, we found that ROS-induced pyroptosis of NSCs by activating the NLRP3/GSDMD pathway. These findings suggest that pyroptosis of NSCs induced by Hb can impede neural regeneration after SAH.

## 1. Introduction

Subarachnoid hemorrhage (SAH) is a critical condition associated with high rates of morbidity and mortality [1]. The mechanisms resulting in poor outcomes in SAH are complex and multifactorial, including early brain injury (EBI) and a host of different ensuing processes, such as delayed cerebral ischemia (DCI) [2]. EBI is therefore considered the primary cause of poor neurological outcomes in SAH patients. Other frequently discussed mechanisms that lead to SAH are either initiated or worsened by hemoglobin (Hb) [3]. Following the lysis of red blood cells in the subarachnoid space, the central nervous system (CNS) is exposed to high levels of Hb as well as its degradation products [4, 5]. Previous studies have reported that cell-free Hb accelerates pathological processes in patients with SAH [6].

Neural stem cells (NSCs) are a group of self-renewing cells that can differentiate into neurons, astrocytes, and oligodendrocytes, which are vital for the formation of neural circuits [7–9]. NSCs are distributed throughout the developing brain and reside in two major neurogenic niches in the adult brain, the subventricular zone (SVZ) of the lateral ventricles and the subgranular zone (SGZ) of the hippocampal dentate gyrus [10, 11]. A previous study showed that the number of NSCs decreased as early as one day after SAH and reached its lowest level at three days after SAH [12]. However, few studies have been reported regarding the effects of Hb on NSCs. We hypothesized that the Hb derived from SAH could affect the homeostasis of NSCs, consequently impairing the plasticity of neural structures and brain function recovery in SAH.

Multiple pathogenic mechanisms involved in the pathophysiology of SAH, including inflammation, oxidative stress, and apoptosis. Oxidative stress is one of the key features of SAH [13]. Accelerated ROS in cells can lead to cell death, including apoptosis, pyroptosis, and necroptosis [14–16]. In addition, previous evidence has indicated that increased formation of ROS can markedly regulate NLRP3 inflammasome and Caspase 1 activation, which plays an essential role in ROS-induced IL-1*β* production [17]. Nonapoptotic inflammatory cell death is increasingly recognized as an important determinant of neurodegeneration. In particular, the proinflammatory and lytic cell death program, known as pyroptosis, has emerged as a key factor in neurological disease and has been implicated in neural disorders ranging from Alzheimer's disease (AD) to traumatic brain injury (TBI) [18–20].

Pyroptosis has been redefined as gasdermin-mediated programmed necrosis and is initiated by Caspase 1 family proteases. Recent studies have shown that the pyroptosis executioner gasdermin D (GSDMD) compromises membrane integrity, resulting in dysfunction of the cell membrane, cell swelling and rupture, and the release of cell contents, interleukin-1*β* (IL-1*β*), and other proinflammatory factors [21, 22]. Inflammasomes have been shown to play an important role in EBI after SAH [23], and the increased expression of NLRP3 has been reported in stroke brains, mainly from neuronal and microglial studies [24]. In this study, the effect and mechanism of Hb on pyroptotic death of NSCs after SAH was investigated.

## 2. Material and Methods

### 2.1. Clinical Sample Collection and IL-1*β* Expression Analysis

The study was approved by the ethics committee of Nanjing Drum Tower Hospital, and written informed consent was obtained from each patient. For SAH clinical and radiographic severity grades, Hunt and Hess (HH) grade and Fisher scale were evaluated on admission. The SAH inclusion criteria were as follows: (1) written informed consent from either the patient or a family member; (2) age ≥ 18 and ≤70 years; and (3) diagnosis of subarachnoid hemorrhage by computed tomography (CT scan). Aneurysms were verified through digital subtraction angiography (DSA). Patients with a history of CNS disease (e.g., CNS infection, stroke, traumatic brain injury, and spinal cord injury) or other organ dysfunctions within the preceding 6 months were excluded. A total of 33 patients with aneurysmal SAH diagnosed by computerized tomography and digital subtraction angiography were enrolled. Cerebrospinal fluid (CSF) samples were collected through lumbar puncture between 24 and 72 h after SAH. For the control group, six patients were admitted to the Department of Neurology as well as Neurosurgery and subjected to lumbar puncture to assist diagnosis. The demographic information of the enrolled subjects is shown in Supplemental Table S1. CSF samples were obtained during spinal anesthesia before surgery as a control group. The levels of IL-1*β* in the CSF were measured by enzyme-linked immunosorbent assay (ELISA) using a commercially available ELISA kit (R&D Systems, #DY201).

### 2.2. Animals and Experimental SAH Model

Adult male Sprague–Dawley rats (250-280 g) were purchased from the Animal Center of Drum Tower Hospital (Nanjing, China). The rats were housed in a temperature (25°C) and humidity-controlled room with a 12 h light/dark cycle. All rats were allowed free access to food and water under controlled humidity and temperature (24 ± 0.5°C) conditions. The experimental protocols and procedures were approved by the Institutional Animal Care and Use Committee at Drum Tower Hospital and conformed to the National Institutes of Health (NIH) Guide for the Care and Use of Laboratory Animals.

The SAH endovascular perforation model in rats was established as previously described [25]. The rats were allowed free access to water, hydrogel, or ground up chow in water after surgery. The SAH grading score was used to estimate the degree of SAH as previously described [26]. A total score ranging from 0 to 18 was obtained by adding the scores of the six segments divided by the basal cistern. SAH grading was performed by a collaborator who was blinded to the experiment.

### 2.3. Primary Neural Stem Cell Culture and Treatment

NSCs were isolated from Sprague–Dawley rats on embryonic Day 14, and the hippocampus was dissected from embryonic brains. The separated blood vessels and meninges from the hippocampus were collected in a Falcon tube and then digested in Accutase (Gibco) for 20 min at 37°C. The Accutase solution was removed, and the cells were gently washed three times with PBS. Dulbecco's modified Eagle's medium (DMEM/F12, Gibco) containing 2% B-27, 20 ng/mL recombinant epidermal growth factor (EGF), 10 ng/mL basic fibroblast growth factor (bFGF), and 1% penicillin–streptomycin supplement were added to the cells. The NSCs were cultured in an incubator at 37°C with 5% CO_2_ gas. To examine the effect of Hb on neural stem cell proliferation, the cells were treated with 25 *μ*M Hb for different amounts of time [27]. For experiments involving pharmacological inhibitors, NSCs were pretreated with 5 mM N-acetyl-L-cysteine (NAC) for 2 h and subsequently treated with Hb for 24 h in the presence of this inhibitor. Hb and NAC were purchased from Sigma–Aldrich (St Louis, MO, USA).

### 2.4. Determination of NSC Proliferation and Cell Survival Curve

Proliferation was determined by a CCK8 (Cell Counting Kit-8) assay and trypan blue staining assay. NSCs (approximately 10,000 cells per well) were seeded into 96-well plates overnight. At different time points posttransfection, Cell CCK8 solution (Beyotime Biotechnology) was added to each well, followed by further incubation. The absorbance was determined at a 450 nm wavelength to assess cell proliferation and cell survival curve, using a microplate absorbance reader (BioTek Instruments). As for trypan blue staining assay, NSCs were treated, prepared, and stained with trypan blue reaction buffer (Sigma-Aldrich, USA). Then, the cells were observed under a light microscope, as the dead cells were counted in blue. Finally, the cell viability was calculated as: cell viability (%) = (total cells − dead blue cells)/total cells × 100%.

### 2.5. LDH Release Assay

After NSCs were treated in accordance with the experimental design described above, and reached confluence, the lactate dehydrogenase (LDH) release was measured using an LDH assay kit (Beyotime Biotechnology). Absorbance was measured with a microplate absorbance reader (BioTek Instruments) at a wavelength of 490 nm. The background optical absorbance was measured at 690 nm and was subtracted from the primary measurements for each well. The fold increase in LDH concentrations was normalized against the control group.

### 2.6. Immunofluorescence Staining

Immunofluorescence (IF) staining was performed according to our previous study [28]. Briefly, brain sections were fixed in 4% paraformaldehyde for approximately 24 h. Coronal paraffin-embedded 4 *μ*m thick slices were processed for antigen retrieval and blocked with 5% BSA for 1 h. After blocking, the slices were incubated overnight at 4°C with the following primary antibodies: mouse anti-nestin (1 : 300, Cell Signaling Technology, #4760); mouse anti-SOX2 (1 : 300, Abcam, #ab79351); rabbit anti-NLRP3 (1 : 400, Biorbyt, #orb1105906); rabbit anti-caspase1 p20 (1 : 400, Biorbyt, #orb454595); and rabbit anti-GSDMD (1 : 400, Biorbyt, #orb1105840). Then, the sections were washed and incubated with the appropriate secondary antibodies.

### 2.7. ELISA

IL-1*β* expression in culture supernatants was detected using an ELISA kit (R&D Systems, #RLB00) according to the manufacturer's instructions. Absorption at 450 nm was determined using a microplate reader (Bio-Rad Laboratories), and the IL-1*β* concentration was determined according to the standard curve generated at the same time.

### 2.8. Assessment of ROS Levels in NSCs

Intracellular ROS levels were determined by flow cytometry (FCM) after staining with 2′,7′-Dichloro-dihydro-fluorescein diacetate (DCFH-DA), purchased from Beyotime Biotechnology. Briefly, cells were labeled with 2.5 *μ*M DCFH-DA for 20 min and analyzed by FCM (BD Biosciences) with reference to the manufacturer's instructions and previous study [29]. The percentage of cells displaying increased dye uptake was used to reflect the increase in ROS levels.

### 2.9. Scanning Electron Microscopy (SEM)

For scanning electron microscopy (SEM), first, the cells were fixed with 2% glutaraldehyde for 24 h and then washed in PBS, and then the cells were postfixed in 1% osmic acid for 2 h at room temperature. Next, the samples were dehydrated in a graded series of ethanol and transferred to isoamylacetate. Finally, the samples were dried in a critical point dryer and imaged with a scanning electron microscope (SU8100, Hitachi).

### 2.10. Quantitative RT–PCR (qRT–PCR)

Total RNA was extracted using TRIzol (Invitrogen). One microgram of RNA was reverse transcribed using the cDNA Archive Kit (Applied Biosystems). Real-time PCR amplification was performed using the primer pairs for individual genes, as shown in Table 1. A 20 *μ*L PCR final reaction volume contained 10 *μ*L of 2X PCR Master Mix (Power SYBR Green kit, Applied Biosystems), 0.4 *μ*L of forward primer, 0.4 *μ*L of backward primer, 8.2 *μ*L of nuclease-free water and 1 *μ*L of cDNA. All reactions were carried out using the same cycling parameters: 95°C for 10 min, 40 three-step cycles of 95°C for 15 s, 60°C for 20 s, and then 72°C for 30 s. Amplification and fluorescence were measured by a StepOnePlus™ System (Applied Biosystems). Changes in the transcript abundance of the tested genes were calculated using the 2^-△△CT^ method.

### 2.11. Western Blot Analysis

Total protein was extracted using the same procedures as described previously [28]. The protein concentrations were determined, and equal amounts of protein lysates were separated by SDS–PAGE and transferred onto polyvinylidene fluoride membranes followed by blocking with 5% skimmed milk. Subsequently, the membranes were incubated with primary antibodies against NLRP3 (1 : 1000, Proteintech, #19771-1-AP), ASC (1 : 500, Santa Cruz, #sc-514414), Caspase1 (1 : 1000, Proteintech, #22915-1-AP), GSDMD (1 : 1000, Cell Signaling Technology, #93709), or GAPDH (1 : 2000, Proteintech, #60004-1-Ig). After washing with TBST, the membranes were incubated with the secondary antibody (1 : 10000). Detection was performed using Immobilon Western Chemiluminescent HRP Substrate (Millipore). The grayscale values of the protein bands were determined using ImageJ software, and then statistical analysis was performed.

### 2.12. Statistical Analysis

All data are expressed as the mean ± SD, and Shapiro–Wilk and Bartlett's tests were performed for normality and homoscedasticity. Statistical comparisons between normally distributed data of the control and experimental groups were analyzed by Student's *t* test and one-way ANOVA followed by Dunnett's multiple comparison test for multiple groups. Statistical analyses were performed using GraphPad Prism 7.0 (GraphPad Software Inc., San Diego, CA, USA) and SPSS 20.0 software (IBM Corp, Armonk, NY), and a value of *p* < 0.05 was considered statistically significant.

## 3. Results

### 3.1. Elevated IL-1*β* Levels in the CSF of Patients with SAH

**Figure 1:**
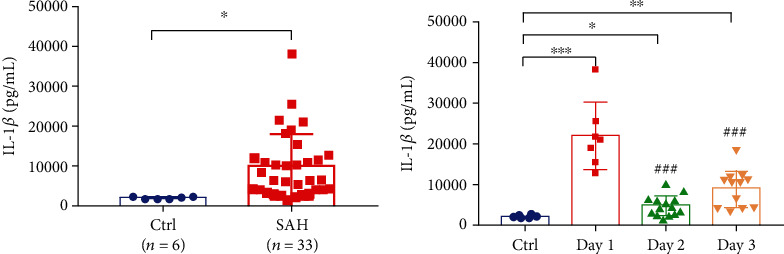
Elevated IL-1*β* levels of CSF in SAH patients. (a) The IL-1*β* of CSF samples in control and SAH patients were determined by ELISA. The mean IL-1*β* level of all CSF samples including day 1-3 after SAH was significantly higher than that of control samples. (b) The IL-1*β* level of CSF in SAH patients at day 1 (*n* = 7) was significantly higher than that at day 2 (*n* = 14) and day 3 (*n* = 12) after SAH. Data were presented as the Mean ± SD. ^∗^*p* < 0.05; ^∗∗^*p* < 0.01; ^∗∗∗^*p* < 0.001 vs. control group; ###*p* < 0.001 vs. SAH at day 1 group.

An elevated level of IL-1*β* is one marker of pyroptosis [21]. The levels of IL-1*β* in the CSF of SAH patients were detected using an ELISA kit. A total of 33 patients who experienced SAH were enrolled in this study, and 6 patients without SAH were included as controls. The levels of IL-1*β* in CSF at day 1, 2, and 3 after the occurrence of SAH were examined. Compared with the non-SAH control samples, the mean level of IL-1*β* in all CSF samples from SAH patients was significantly increased (Figure 1(a)). To investigate the dynamic change in IL-1*β*, the IL-1*β* levels in the CSF of SAH patients were analyzed on day 1, 2, and 3. The CSF IL-1*β* levels in SAH patients were significantly higher than those in control patients at all time points assessed. The level of IL-1*β* in the CSF of patients in the acute phase of SAH (at day 1) was markedly higher than that at day 2 and Day 3 (*p* < 0.05) (Figure 1(b)).

### 3.2. NLRP3 Inflammasome-Mediated Pyroptosis in the Hippocampus after SAH

**Figure 2:**
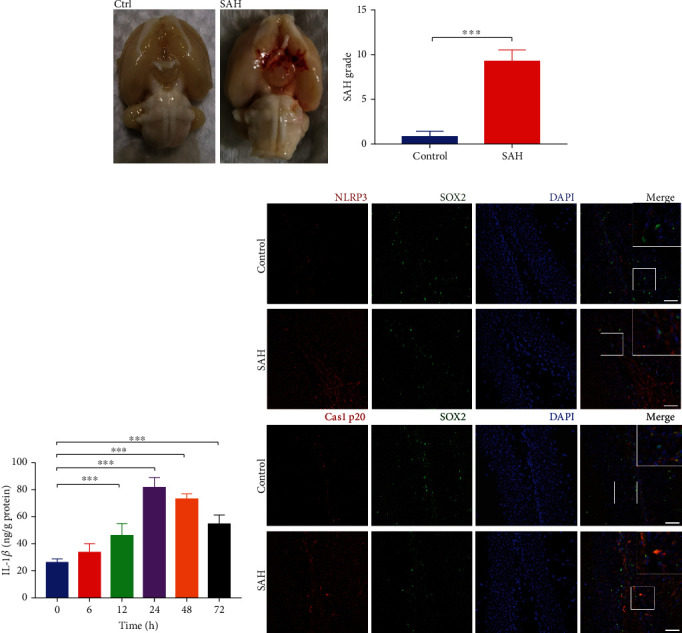
NLRP3 mediated pyroptosis in rat hippocampus after SAH. (a) Representative images of the rat brains in control and SAH group after operation. (b) The SAH grade scores of the rats in control and SAH groups. (c) IL-1*β* expression in hippocampus were detected by ELISA. (d) Representative IF images of NLRP3 and Caspase1 p20 in hippocampus of rats after SAH. Scale bar = 50 *μ*m. Data were expressed as Mean ± SD (*n* = 6), ^∗∗∗^*p* < 0.001 vs. control group.

A total of 30 rats were used in this study and were divided into a sham-operated control group (8 rats) and an SAH model group (22 rats). The mortality was 18.2% (4 of 22) in the SAH group and 0.0% (0 of 8) in the control group. Blood clots, which were mainly distributed around the Circle of Willis, were scored at 72 h (Figure 2(a)). The average SAH grade in the SAH group was 9 at 72 h after SAH (Figure 2(b)), which was significantly higher than that in the control group, indicating that the SAH model was successful. SAH also induced a significant increase in IL-1*β* compared with the control (Figure 2(c)). Caspase1 p20 is a marker of pyroptosis in cells. Immunofluorescence staining showed that the expression of NLRP3 and Caspase1 p20 colocalized with SOX2, a marker of NSCs in the rat hippocampus, was clearly increased in the hippocampus at 24 h after SAH compared with the control group, indicating that SAH induced pyroptosis of NSCs in the hippocampus (Figure 2(d)).

### 3.3. NLRP3/GSDMD Mediated Pyroptosis in NSCs In Vitro

**Figure 3:**
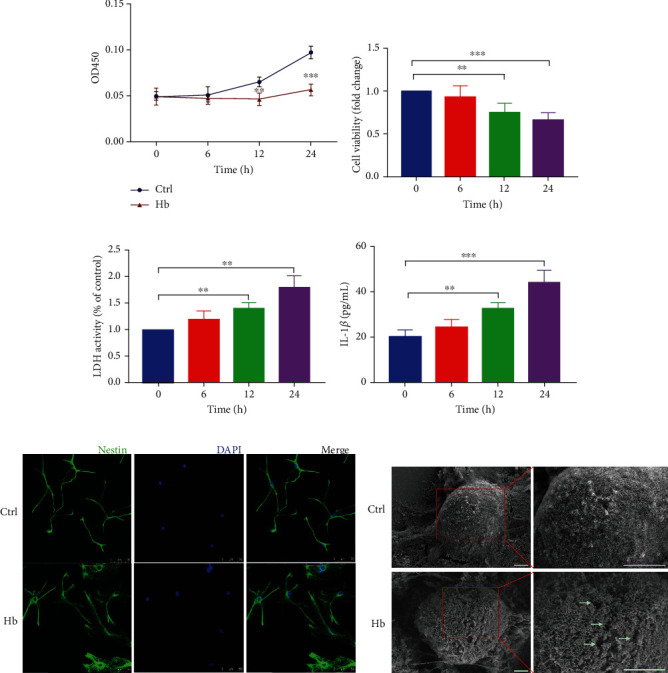
Hb triggered pyroptosis in primary NSCs in vitro. (a–c) Cell survival curve (a), cell viability (b), and LDH release (c) of primary NSCs treated by 25 *μ*M Hb at different time points. (d) IL-1*β* protein secreted in the supernatant of NSCs after Hb treatment using ELISA. (e) Representative images of Nestin immunofluorescence staining in NSCs, which displayed pyroptotic typical swelling at 24 h after Hb treatment. Scale bar = 50 *μ*m. (f) Representative scanning electron microscopy (SEM) images of NSCs at 24 h showed more pore formations on membrane on NSCs as white arrows in Hb treatment group. Scale bar = 2 *μ*m. Data were expressed as Mean ± SD (*n* = 4), ^∗∗^*p* < 0.01, ^∗∗∗^*p* < 0.001 vs. control group.

After finding that SAH can induce elevated expression of NLRP3 and IL-1*β* in the hippocampus and pyroptosis of endogenous hippocampal NSCs, the primary NSCs from rat hippocampus were isolated to investigate the effect of Hb, the main component of SAH, on NSCs. The primary NSCs were identified by immunostaining against the NSC marker Nestin (Supplementary Figure S1). First, the CCK-8 assay, trypan blue staining assay and LDH release assay were used to test the effects of Hb on the cell viability and survival curve of NSCs. The results showed that Hb treatment significantly inhibited the proliferation of NSCs at first 24 h (Figures 3(a)–3(b)) and triggered cell death as treated more than 3 days (Supplementary Figure S2), and enhanced the LDH activity of NSCs in a time-dependent manner (Figure 3(c). Consistent with the in vivo results, Hb also significantly elevated the levels of IL-1*β* in NSCs in a time-dependent manner (Figure 3(d)).

As previously reported, pyroptotic death is distinguished from other forms of cell death partly due to its unique morphological and biochemical characteristics, such as cellular swelling and pore formation of the cell membrane [19, 20]. As shown in Figure 3(e), IF staining of Nestin displayed pyroptotic typical swelling in Hb-treated NSCs. In addition, the changes in the plasma membrane of NSCs after Hb treatment were investigated by scanning electron microscopy (SEM). Compared with the control group, a large number of membrane pores were formed on the NSCs after Hb treatment, while few membrane pores were observed on the untreated NSCs. (Figure 3(f)). These results indicated that Hb mediated inhibition of NSC viability may be due to pyroptosis.

### 3.4. Hb Treatment Activates the NLRP3/GSDMD Signaling Pathway in NSCs

**Figure 4:**
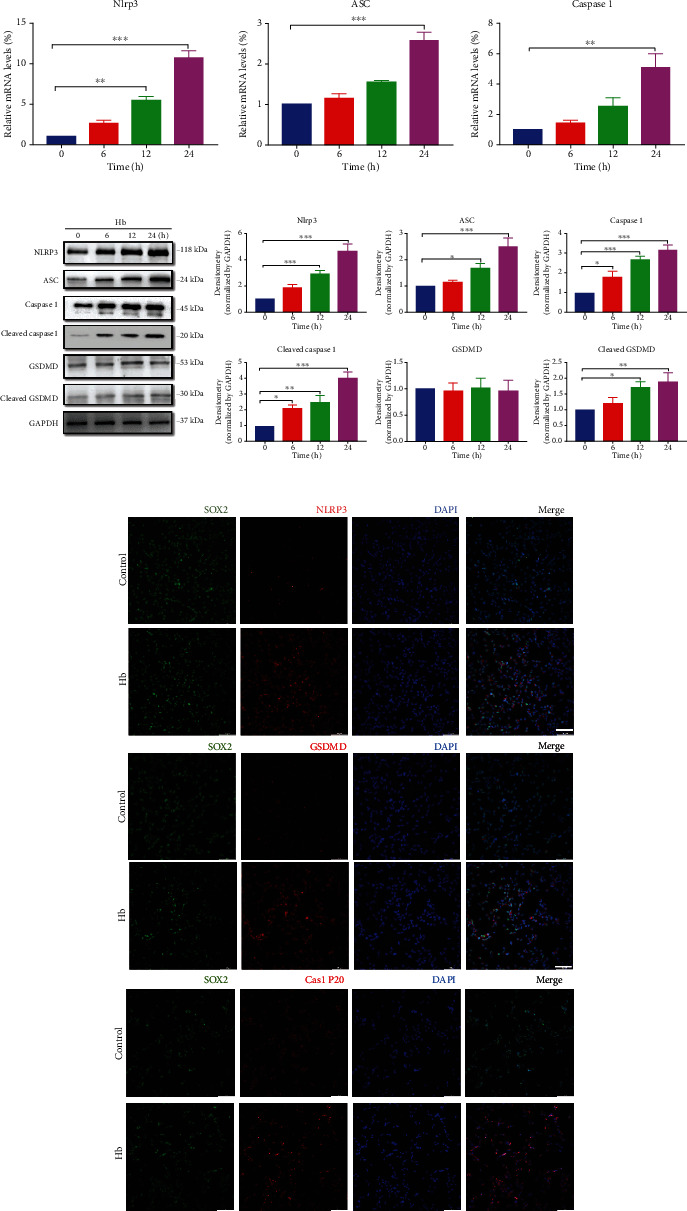
The NLRP3/GSDMD mediated the pyroptosis in primary NSCs exposed to Hb. (a) Hb treatment induced the elevated mRNA expressions of NLRP3, ASC, and Caspase1 in NSCs using qRT-PCR assay. (b) Western blot analysis showed the upregulated protein expressions of NLRP3, ASC, Cleaved Caspase1, and Cleaved GSDMD in Hb-treated NSCs. The band densities in western blot analysis were quantified using Image J Software. (c) Representative IFs staining images of NLRP3,Caspase1 P20, and GSDMD in NSCs with or without Hb treatment at 24 h. Scale bars = 50 *μ*m. Data were expressed as Mean ± SD (*n* = 4), ^∗^*p* < 0.05, ^∗∗^*p* < 0.01, ^∗∗∗^*p* < 0.001 vs. control group.

Next, western blotting, qRT–PCR, and IF staining were performed to assess the expression of NLRP3 and pyroptosis-related proteins, including ASC, GSDMD, GSDMD-N (cleaved GSDMD), Caspase1, and Caspase1 p20 (cleaved Caspase1), after Hb treatment. The mRNA levels of pyroptosis-related genes in NSCs were markedly enhanced after Hb treatment compared with the control group (Figure 4(a)). As shown (Figure 4(b)), the protein expression levels of NLRP3, ASC, cleaved Caspase1, and cleaved GSDMD were weakly expressed in untreated NSCs but were significantly increased after Hb treatment in a time-dependent manner. Consistent with the western blot analysis, NLRP3, Caspase1 P20, and GSDMD were weakly stained in untreated NSCs but were markedly enhanced after Hb treatment (Figure 4(c)).

Taken together, these results suggest that Hb induces pyroptosis in NSCs after SAH via activation of the NLRP3/GSDMD pathway.

### 3.5. Effects of ROS on Hb-Induced Pyroptosis via the NLRP3/GSDMD Pathway

**Figure 5:**
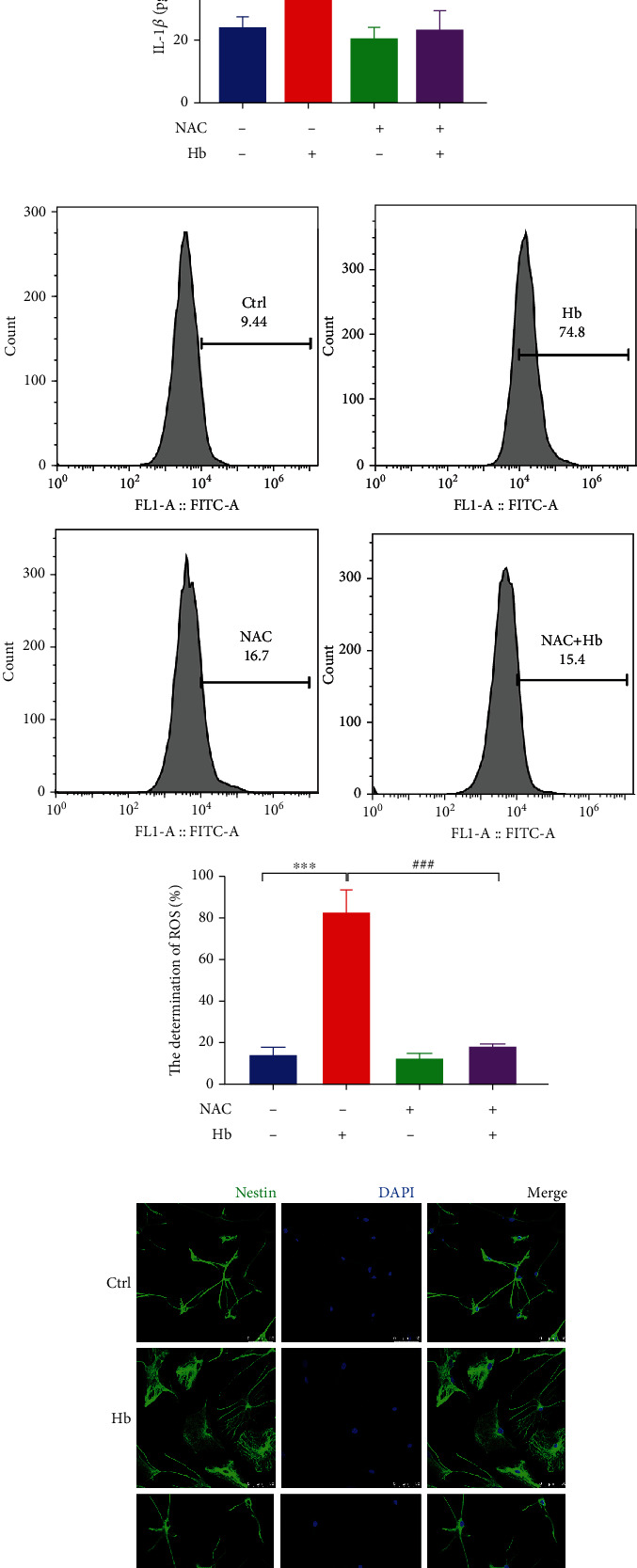
Effects of ROS on Hb-induced pyroptosis in primary NSCs. (a) Hb treatment significantly induced ROS production in intracellular of primary NSCs by FCM analsysis. (b) The Hb-induced inhibition of cell viability in NSCs was retrieved by NAC pretreatment (a ROS inhibitor). (c) NAC prestreatment restored Hb-induced LDH release in NSCs. (d) Secreted IL-1*β* in the supernatant was decreased in NSCs when pretreated with NAC. (e) NAC pretreatment blocked the Hb-induced ROS production in intracellular of primary NSCs. (f) Representative images Nestin immunofluorescence stainingin NSCs and the cell body area was analyzed using Image J Software. Scale bar = 50 *μ*m. Data were expressed as Mean ± SD (*n* = 3). ^∗∗^*p* < 0.01, ^∗∗∗^*p* < 0.001 compared with the control group. #*p* < 0.05, ###*p* < 0.001 vs. Hb treatment group.

As ROS are essential for inflammasome activation, changes in ROS levels were examined to elucidate the possible role of ROS in Hb-induced pyroptosis of NSCs. Flow cytometry analysis showed that the ROS level in NSCs was markedly enhanced after Hb treatment, indicating that Hb treatment induced ROS accumulation in NSCs (Figure 5(a)). Hb-induced ROS production was clearly inhibited when NSCs were cultured with NAC, a ROS inhibitor (Figure 5(e)). NAC was used to further confirm that ROS accumulation was involved in Hb-induced pyroptosis in NSCs. The results showed that NAC pretreatment markedly reversed the Hb-induced decrease in cell viability (Figure 5(b)) and decreased Hb-induced LDH release in NSCs (Figure 5(c)). In addition, elevated IL-1*β* expression was also blocked by NAC pretreatment in NSCs (Figure 5(d)). NAC pretreatment also suppressed pyroptotic typical swelling induced by Hb treatment in NSCs (Figure 5(f)), implicating ROS as one of the main regulators of pyroptosis in response to Hb in NSCs.

### 3.6. ROS Regulate the NLRP3/GSDMD Pathway in Hb-Induced Pyroptosis of NSCs

**Figure 6:**
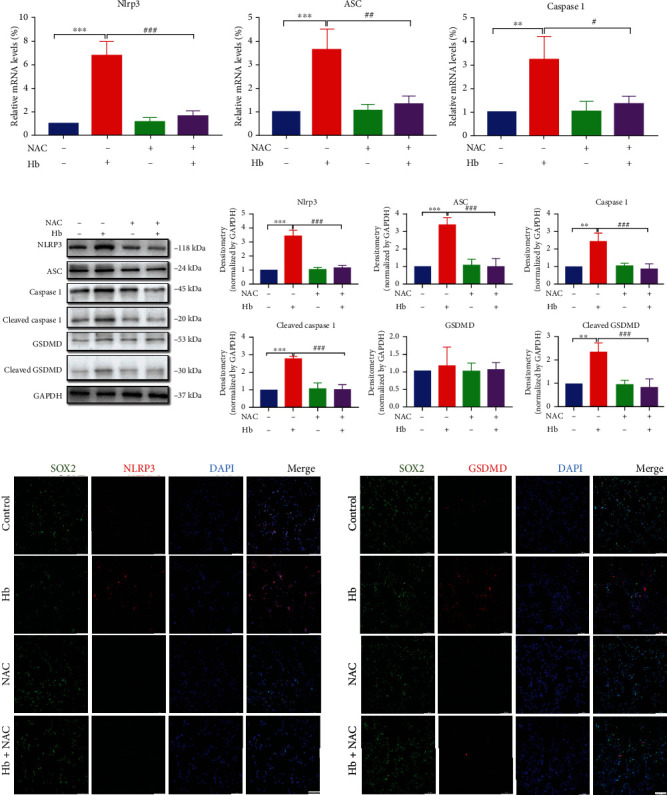
ROS scavenger mitigated activation of NLRP3/GSDMD pathway in Hb-induced pyroptosis of NSCs. (a) Hb-induced upregulated mRNA levels of NLRP3, ASC, and Caspase 1 were restored by NAC pretreatment by qRT-PCR assay (b) Western bolt analysis showed that NAC pretreatment blocked the upregulated protein levels of NLRP3, Caspase 1, Cleaved Caspase 1, Cleaved GSDMD, and ASC by Hb in NSCs. (c) Representative images of NLRP3 and GSDMD immunostainings in NSCs. Scale bars = 50 *μ*m. Data were expressed as Mean ± SD (*n* = 3).^∗∗^*p* < 0.01, ^∗∗∗^*p* < 0.001 compared with the control group. #*p* < 0.05, ###*p* < 0.001 vs. Hb treatment group.

Finally, we investigated whether ROS regulated NLRP3/GSDMD pathway activation in Hb-induced pyroptosis of NSCs. After ROS production in NSCs was inhibited by NAC, qRT–PCR, western blot analysis, and IF staining were performed to investigate NLRP3/GSDMD pathway activation by Hb treatment. The results showed that NAC markedly alleviated Hb-induced mRNA expression of inflammasome components, including NLRP3, ASC, and Caspase 1 (Figure 6(a)), and the Hb-induced elevated protein levels of NLRP3, ASC, cleaved Caspase 1, and cleaved GSDMD in NSCs (Figure 6(b)). Moreover, IF staining also showed that Hb-induced NLPR3 and GSDMD expression was also significantly reduced in NSCs after NAC treatment (Figure 6(c)). Thus, these results clearly show that ROS regulates NLRP3/GSDMD pathway activation in Hb-induced pyroptosis of NSCs.

## 4. Discussion

**Figure 7:**
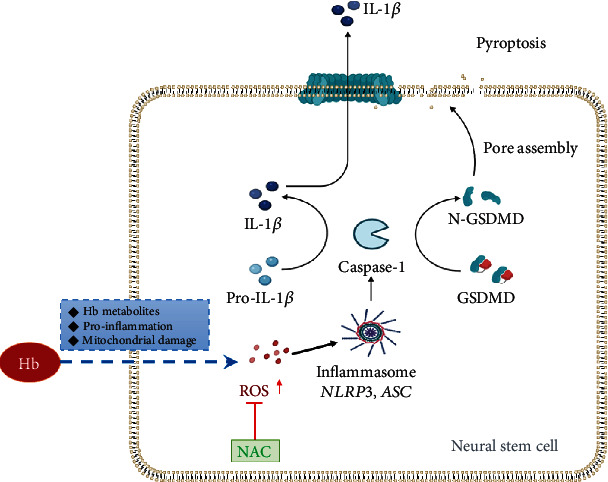
Proposed model of pyroptosis induced by hemoglobin via ROS/NLRP3 axis in NSCs.

In the present study, the possible role of ROS-induced pyroptosis of NSCs mediated by the NLRP3 inflammasome in the pathogenesis of SAH was investigated. Our results showed that levels of IL-1*β* in the CSF of SAH patients were significantly higher than those in control patients. In SAH patients, the NLRP3 inflammasome mediated pyroptosis of NSCs in the hippocampus. Hb derived from subarachnoid hemorrhage triggered pyroptosis of primary NSCs in vitro, resulting in cellular swelling, pore formation of the cell membrane, and the expression of pyroptosis-related proteins. Next, we further showed that the ROS/NLRP3/GSDMD pathway mediated Hb-induced pyroptosis of NSCs (Figure 7).

SAH is a type of hemorrhagic stroke usually caused by a ruptured cerebral aneurysm and leads to poor clinical outcomes due to its significant morbidity and mortality. One important reason is that lost or dead neurons cannot be replaced by new ones differentiated from endogenous NSCs. In recent years, stem cells have gained interest because of their ability to extensively proliferate, self-renew, and differentiate into different types of cells and tissues, offering the possibility to treat multiple diseases and disorders. The existence of NSCs in the adult brain represents a promising avenue of therapy for the replacement of lost neurons from the deleterious effects of SAH.

It is well known that the mature human central nervous system (CNS) shows little potential for regeneration. NSCs are self-renewing and multipotent cells that hold the potential to differentiate into multiple cell lineages, such as neurons, astrocytes, and oligodendrocytes. In adults, NSCs mainly persist in the SVZ and the subgranular zone (SGZ) of the dentate gyrus (DG), which can serve as a source for cell replacement of damaged neurons [30]. According to different sites of occurrence, neural regeneration can be divided into central nervous system (CNS) neural regeneration and peripheral nervous system (PNS) neural regeneration [31]. NSC transplantation can be used to recover function from neurological disorders, such as spinal cord injury [32] and cerebral infarction [33]. Furthermore, researchers have found that NSCs can differentiate into Schwann cells (SCs) and secrete neurotrophic factors for nerve growth in the PNS [31].

Cell replacement therapy involves the transplantation of NSCs and is a promising regenerative strategy for the repair of CNS damage. However, these endogenous NSCs remain largely quiescent. These cells can give rise to intermediate progenitor cells and then progressively differentiate into mature neurons through the neuroblast phase [34]. Following brain injury or infection, cytokines and other factors are released in situ, which can strongly influence NSC activity [35]. Until now, it has remained elusive whether the released Hb and increased inflammatory cytokines might have adverse effects on the biological activity of NSCs after SAH, thus leading to poor outcomes.

Pyroptosis is a new paradigm of cell death that occurs with unique features, including cell swelling and membrane pore formation that compromise membrane integrity [36, 37]. Pyroptosis is related to multiple diseases and can be involved in a variety of acute and chronic injuries. During progression of pyroptosis, activated Caspase 1 triggers the maturation of the proinflammatory cytokines IL-1*β* and IL-18, ultimately leading to pyroptotic cell death. This finding is consistent with the results of the present study, including typical cell swelling and pore formation in the NSC membrane after Hb treatment. In addition, we found that levels of IL-1*β*, a typical characteristic of pyroptosis, were significantly increased in the CSF of SAH patients compared with the control cohort. In the SAH model in vitro, we also found that the secretion of IL-1*β* was increased in Hb-treated NSCs. Many studies have demonstrated that neuroinflammation contributes to the pathogenesis of early brain injury induced by SAH, indicating that IL-1*β* plays an important role in the pyroptosis of NSCs after SAH.

Pyroptosis is initiated by Caspase-1 family proteases, which are activated on cytosolic multiprotein platforms termed inflammasomes [38, 39]. The NLRP3 inflammasome has been reported to play an important role in inflammation-associated acute CNS diseases, such as SAH [40–44]. The NLRP3 signaling pathway participates in almost all well-known mechanisms of cell death, including pyroptosis [43, 45–47]. GSDMD is a substrate of Caspase-1, and its cleavage at the predicted site (N-terminal gasdermin domain and GSDMD-N) is required for pyroptosis during inflammasome activation [48]. A previous study demonstrated that GSDMD-induced neuronal cell pyroptosis mediated by the AIM2 inflammasome might be involved in EBI following SAH [49]. It was also reported that minocycline or atorvastatin treatment could markedly alleviate EBI by preventing NLRP3 inflammasome-induced inflammation and pyroptosis [50, 51]. In this study, we also found that the expression of ASC was increased, Caspase-1 signaling was activated, and GSDMD production was enhanced following activation of the NLRP3 inflammasome in NSCs after SAH.

Increasing evidence has demonstrated that ROS plays an important role in brain injury after SAH. Hb metabolite axis of hemoglobin-heme-iron might be the key contributor to ROS production after intracerebral hemorrhage, unstable extracellular hemoglobin makes its heme groups spontaneously oxidize to ferric methemoglobin, and release a superoxide in the reaction [52, 53]. Furthermore, the other factors, such as proinflammatory effect and mitochondrial damage, are also involved in the Hb induced ROS accumulation [52, 53]. ROS have been proposed to promote deubiquitylation of NLRP3 and then activate the NLRP3 inflammasome [54].

Our results showed that ROS overproduction was promoted by Hb in NSCs and that this oxidative stress may induce the activation of the NLRP3 inflammasome, which can be neutralized by N-acetyl-L-cysteine (NAC), a ROS inhibitor. As an antioxidant agent, NAC attenuated both inflammasome activation and inflammatory cytokine generation, as well as the upregulated proteins involved in pyroptosis, such as GSDMD-N and ASC, suggesting that GSDMD cleavage occurrs downstream of ROS release. Subsequently, the pyroptotic death induced by Hb in NSCs was prevented by NAC. These results demonstrate that NLRP3 inflammasome-mediated pyroptosis was significantly alleviated in NSCs following ROS reduction. In addition, several studies have demonstrated that ROS generation induces NLRP3 inflammasome-dependent pyroptotic cell death [14, 16, 55], and Wu et al. reported that nicotine promotes atherosclerosis via ROS/NLRP3-mediated endothelial cell pyroptosis [56]. Other studies have reported the protective effects of antioxidants, such as metformin, on cell pyroptosis via the NLRP3/GSDMD pathway [57]. Thus, ROS accumulation may be the trigger that induces NLRP3/GSDMD-mediated pyroptosis in NSCs after SAH.

However, there were also some limitations in this study. Firstly, the clinical sample size was small, and more samples should be collected to investigate the role of pyroptosis in SAH patients. The precise mechanism by which Hb induces the elevated ROS production in NSCs was not demonstrated clearly in this study. However, it has been shown that mitochondria play an important role in the generation of ROS. Therefore, the role of mitochondria would be explored in future studies. In addition, although NAC was found to inhibit ROS and reverse Hb damage in NSCs, it is still necessary to explore the effect of antioxidant drugs on the treatment of SAH.

In summary, our findings show that pyroptosis is likely a cellular mechanism underlying the detrimental effects of Hb on NSCs through the production of ROS and activation of NLRP3/GSDMD after SAH, which provides a novel therapeutic candidate for the regeneration of NSCs after SAH.

## Figures and Tables

**Table 1:** List of primers used in this study.

Primers	Forward sequence (5′ to 3′)	Reverse sequence (5′ to 3′)
NLRP3	ACTTCTGCACCCCGACTGTA	GAGCGTCACCACACACAGAT
ASC	GAGTCTGGAGCTGTGGCTACTG	ATGAGTGCTTGCCTGTGTTGGT
Caspase1	TCGGAGAGTCGGAGCTGATGTT	CTCTGGGCAGGCAGCAAATTCT
*β*-Actin	GAACCCTAAGGCCAACCGTG	AACCGCTCATTGCCGATAGT

## Data Availability

The data analyzed in the current study are available from the corresponding author on reasonable request.

## Abbreviations

AD: Alzheimer's disease
ASC: Apoptosis-associated speck-like protein containing a card
bFGF: Basic fibroblast growth factor
CCK-8: Cell counting kit-8
CSF: Cerebrospinal fluid
DCFH-DA: 2′,7′-Dichloro-dihydro-fluorescein diacetate
EBI: Early brain injury
EGF: Epidermal growth factor
ELISA: Enzyme-linked immunosorbent assay
FCM: Flow cytometry
GSDMD: Gasdermin d
Hb: Hemoglobin
IL-1*β*: Interleukin-1*β*
LDH: Lactate dehydrogenase
NLRP3: Nod-like receptor thermal protein domain associated protein 3
NSCs: Neural stem cells
qRT-PCR: Quantitative reverse transcription-polymerase chain reaction
ROS: Reactive oxygen species
SAH: Subarachnoid hemorrhage
SEM: Scanning electron microscopy
SGZ: Subgranular zone
SVZ: Subventricular zone
TBI: Traumatic brain injury
IF: Immunofluorescence
NAC: N-acetyl-cysteine
PBS: Phosphate buffer saline.

## Supplementary Materials

Supplementary 1 Supplementary Figure S1. Determination of neurosphere and neural stem cells (NSCs). (A) Representative images of cultured neurospheres. (B) The NSCs was identified by Nestin immunostaining (*n* = 3).

Supplementary 2 Supplementary Figure S2. Cell viability of primary NSCs treated by 25 *μ*M Hb at different time points. (A–E) Cell viability was examined by trypan blue staining assay, as NSCs was treated by Hb from Day 0 to Day 7, respectively. (F) Cell viability decreased significantly after Hb treatment. Data were expressed as Mean ± SD (*n* = 3), ^∗∗∗^*p* < 0.001 vs. Day 0.

Supplementary 3 Table S1. Patient characteristics and clinical features.
