# Supplementary figures and images for: Hemoglobin Derived from Subarachnoid Hemorrhage-Induced Pyroptosis of Neural Stem Cells via ROS/NLRP3/GSDMD Pathway
Source: Oxid Med Cell Longev. 2023 Jan 16;2023:4383332. doi: 10.1155/2023/4383332 (PMC9871413; doi:10.1155/2023/4383332)

A

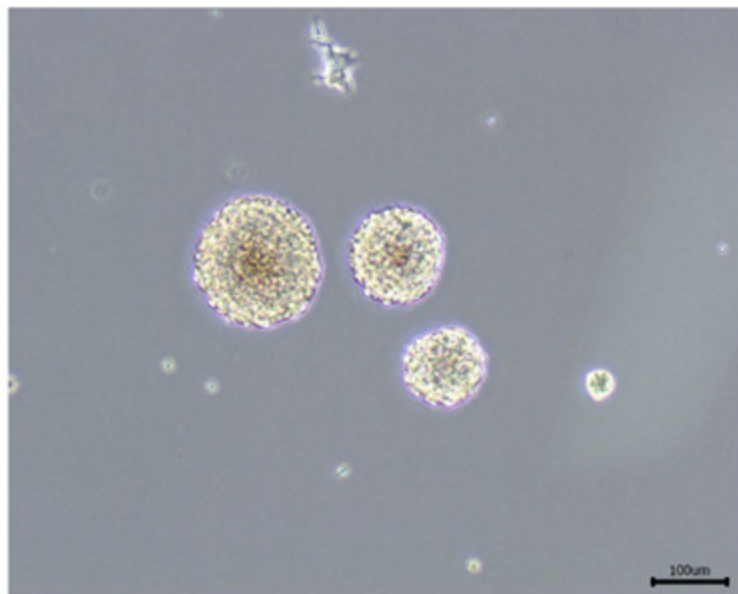

B

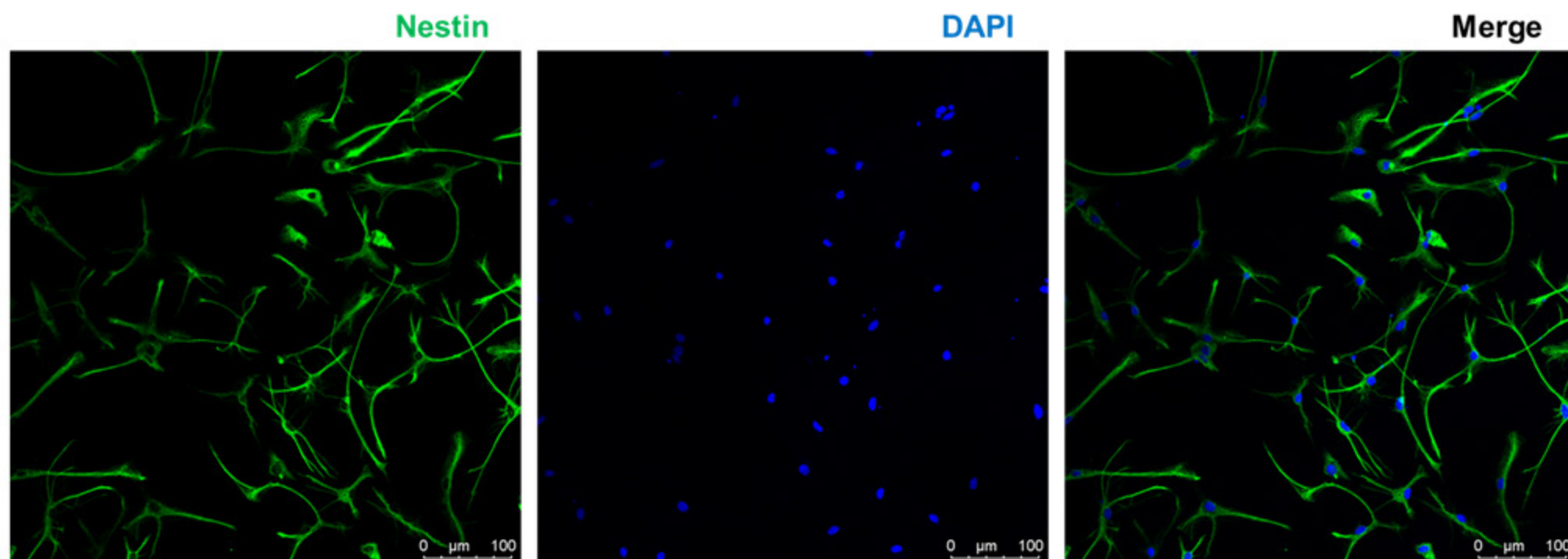

Supplement: Supplementary 1 — Supplementary Figure S1. Determination of neurosphere and neural stem cells (NSCs). (A) Representative images of cultured neurospheres. (B) The NSCs was identified by Nestin immunostaining (n = 3). [file 4383332.f1.pdf]

A

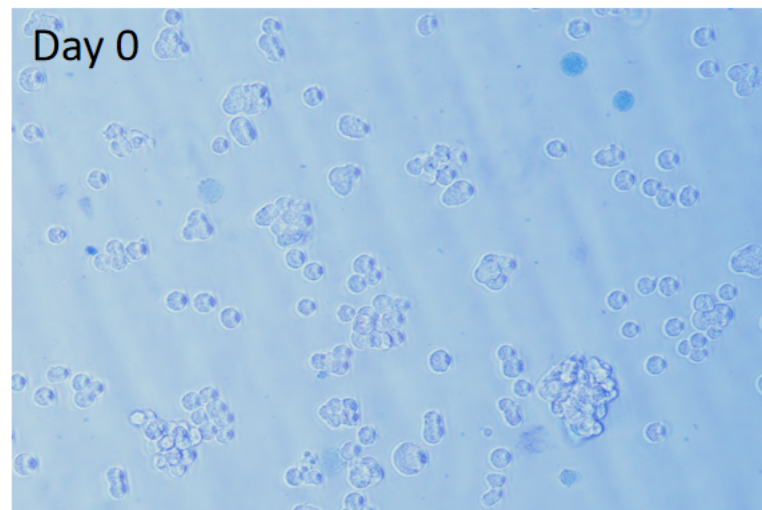

B

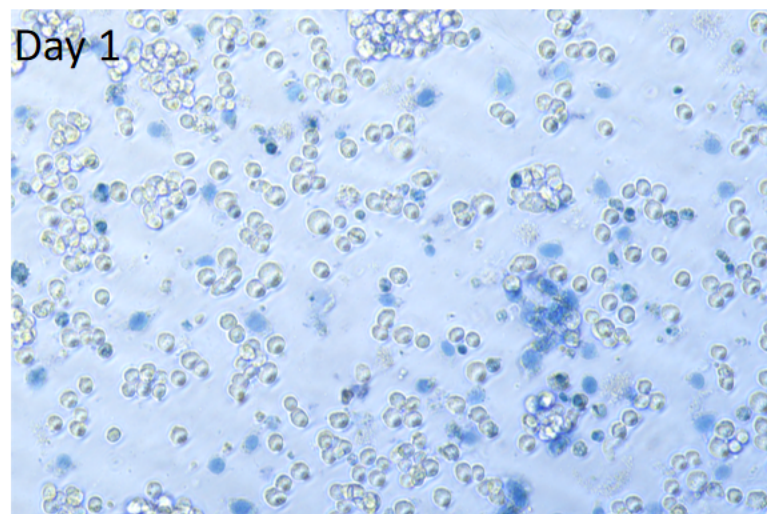

C

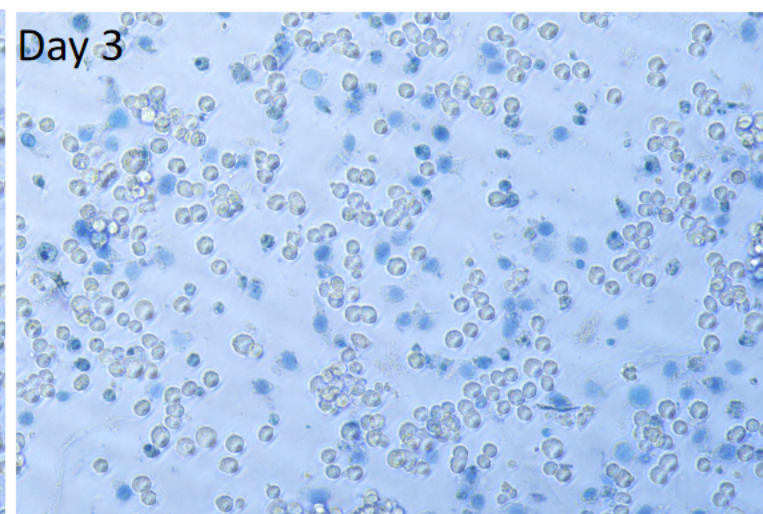

D

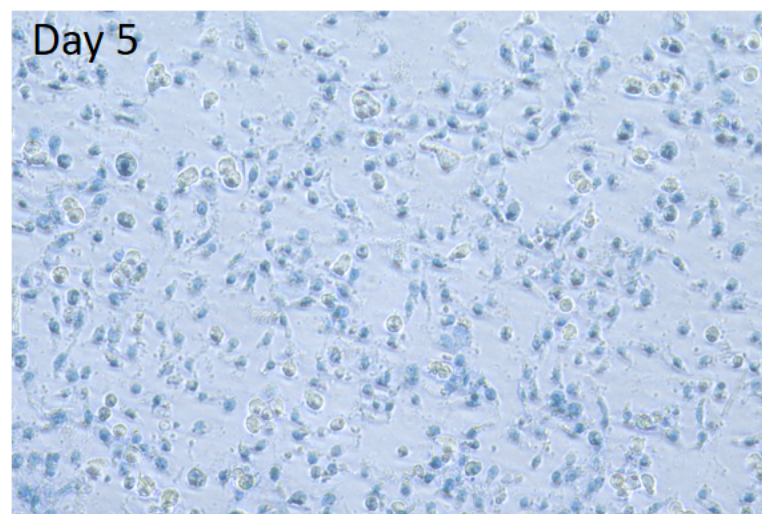

E

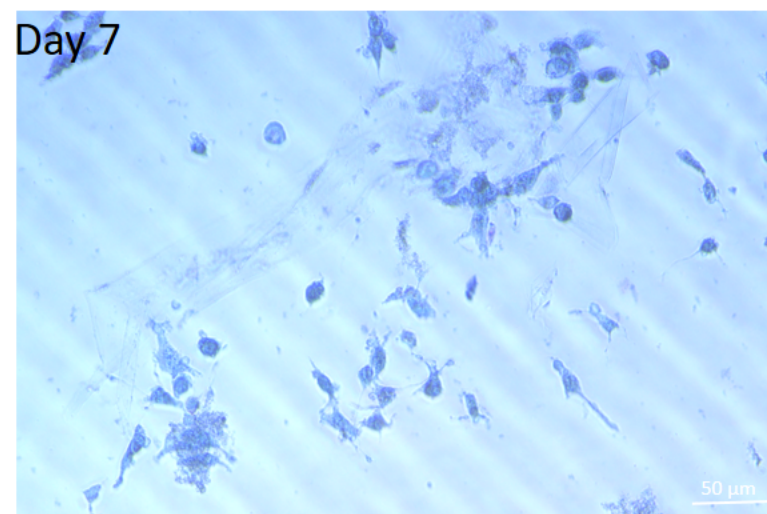

F

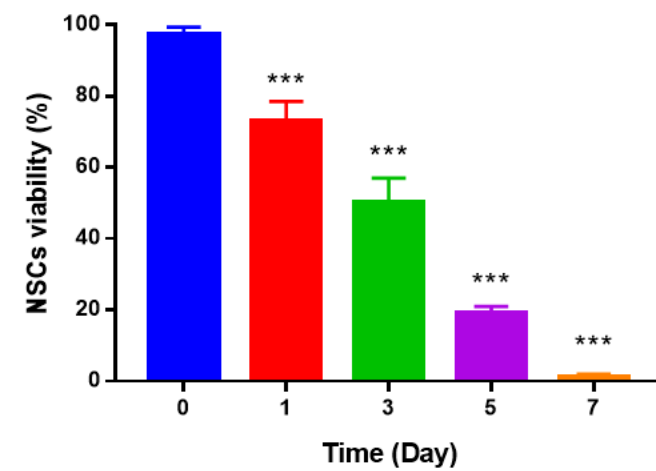

Supplement: Supplementary 2 — Supplementary Figure S2. Cell viability of primary NSCs treated by 25 μM Hb at different time points. (A–E) Cell viability was examined by trypan blue staining assay, as NSCs was treated by Hb from Day 0 to Day 7, respectively. (F) Cell viability decreased significantly after Hb treatment. Data were expressed as Mean ± SD (n = 3), ∗∗∗p < 0.001 vs. Day 0. [file 4383332.f2.pdf]
