# Supplementary material for: Hemoglobin Derived from Subarachnoid Hemorrhage-Induced Pyroptosis of Neural Stem Cells via ROS/NLRP3/GSDMD Pathway
Source: Oxid Med Cell Longev. 2023 Jan 16;2023:4383332. doi: 10.1155/2023/4383332 (PMC9871413; doi:10.1155/2023/4383332)
Supplement: Supplementary 3 — Table S1. Patient characteristics and clinical features. [file 4383332.f3.docx]

|  | Control (n=6) | SAH (n=33) | P | |
| --- | --- | --- | --- | --- |
| Age (years, mean±SD) | 58.2±12.1 | 61.6±9.1 | 0.421 | |
| Gender n(%) |  |  |  | |
| male | 3 (50) | 13 (39) | 0.627 | |
| female | 3 (50) | 20 (61) |  | |
| Location of aneurysm n(%) |  |  |  | |
| anterior communicating artery |  | 10 (30) |  | |
| basilar artery |  | 2 (6) |  | |
| internal carotid artery |  | 3 (9) |  | |
| middle cerebral artery |  | 2 (6) |  | |
| posterior communicating artery | | 14 (42) |  |  |
| posterior inferior cerebellar artery | | 1 (3) |  |  |
| superior cerebellar artery |  | 1 (3) |  | |
| Hunt−Hess Scale n(%) |  |  |  | |
| I |  | 3 (9) |  | |
| II |  | 12 (36) |  | |
| III |  | 11 (33) |  | |
| IV |  | 7 (21) |  | |
| V |  | 0 (0) |  | |
| Fisher grade n(%) |  |  |  | |
| 1 |  | 1 (3) |  | |
| 2 |  | 6 (18) |  | |
| 3 |  | 19 (58) |  | |
| 4 |  | 7 (21) |  | |

Table S1 Patient characteristics and clinical features
